# Characterization of the Chitinase Gene Family in Mulberry (*Morus notabilis*) and *MnChi18* Involved in Resistance to *Botrytis cinerea*

**DOI:** 10.3390/genes13010098

**Published:** 2021-12-31

**Authors:** Youchao Xin, Donghao Wang, Shengmei Han, Suxia Li, Na Gong, Yiting Fan, Xianling Ji

**Affiliations:** 1 College of Forestry, Shandong Agricultural University, Tai’an 271018, China; ycxin@sdau.edu.cn (Y.X.); wangdh1204@163.com (D.W.); gnsmooth@163.com (N.G.); 2 State Key Laboratory of Crop Biology, Shandong Agricultural University, Tai’an 271018, China; hanmei203203@163.com (S.H.); LI152153@163.com (S.L.); fan18303215087@163.com (Y.F.)

**Keywords:** chitinase, *B. cinerea*, mulberry, *MnChi18*

## Abstract

Chitinase is a hydrolase that uses chitin as a substrate. It plays an important role in plant resistance to fungal pathogens by degrading chitin. Here, we conducted bioinformatics analysis and transcriptome data analysis of the mulberry (*Morus notabilis*) chitinase gene family to determine its role in the resistance to *Botrytis cinerea*. A total of 26 chitinase genes were identified, belonging to the GH18 and GH19 families. Among them, six chitinase genes were differentially expressed under the infection of *B. cinerea*. *MnChi18*, which significantly responded to *B. cinerea*, was heterologously expressed in *Arabidopsis* (*Arabidopsis thaliana*). The resistance of *MnChi18* transgenic *Arabidopsis* to *B. cinerea* was significantly enhanced, and after inoculation with *B. cinerea*, the activity of catalase (CAT) increased and the content of malondialdehyde (MDA) decreased. This shows that overexpression of MnChi18 can protect cells from damage. In addition, our study also indicated that *MnChi18* may be involved in *B. cinerea* resistance through other resistance-related genes. This study provides an important basis for further understanding the function of mulberry chitinase.

## 1. Introduction

Plants have several defense mechanisms to resist the invasion of pathogens, including pathogenesis-related (PR) proteins. Chitin is an insoluble polymer, β-1,4-linked N-acetyl-D-glucosamine. It is an important component of the cell wall of pathogenic fungi, but it does not exist in plants. Chitinase (EC 3.2.1.14), a subgroup of PR proteins [1], exists in a variety of organisms and catalyzes the hydrolysis of the β-1-4-linkage in the N-acetyl-D-glucosamine polymer of chitin. The resulting chitin fragments act as powerful pathogen-associated molecular patterns (PAMPs) that induce PAMP-triggered immunity [2,3]. Therefore, chitinase is considered to be a defense-related gene against pathogens containing chitin. Plant chitinases are divided into PR-3, PR-4, PR-8 and PR-11 [4]. Some studies have shown that the increase in chitinase levels is a response to pathogen attack [5,6,7,8,9]. Chitinases are either directly induced by pathogen elicitors or are constitutively expressed in the attacked tissue [10,11]. Chitinases isolated from plants can limit the growth of chitin-containing fungi in vitro [12,13] and in vivo [14], and the overexpressed chitinases in plants can resist infection by different fungal pathogens [15,16,17,18,19].

According to the similarity of amino acid sequence in the catalytic domain, chitinases can be divided into glycosyl hydrolases families 18 and 19 (GH18 and GH19). According to their phylogeny, catalytic reaction mechanism, three-dimensional (3D) structure and sensitivity to inhibitors, these families are further divided into five different classes (Classes I–V). GH18 chitinases (Classes III and V) are widely distributed in various organisms, while GH19 chitinases (Classes I, II and IV) mainly exist in plants and are the main source of chitinolytic activity [20].

Mulberry (*Morus notabilis*) is a typical perennial woody plant with very important economic and medicinal value, because mulberry contains abundant secondary metabolites beneficial to human health [21,22,23,24]. *B. cinerea* is a necrotizing fungal pathogen that can infect more than 200 plant species in the world, including important economic horticultural crops [25,26,27]. *B. cinerea* is also one of the main pathogens affecting mulberry [28]. So far, there are only a few reports of mulberry genes that are effective against *B. cinerea* [28,29]. Plant chitinases resist fungal infection by producing hypersensitivity reactions and inducing defense reactions. Therefore, chitinase is a good target for studying the defense response to *B. cinerea*. However, so far, the role of mulberry chitinase genes in resistance has not been systematically studied.

The availability of the mulberry genome and transcriptome data in response to *B. cinerea* infection has facilitated the identification of genome-wide chitinase gene families and the study of their resistance to *B. cinerea* infection [29,30]. This study reports the genome-wide identification and analysis of the mulberry chitinase gene family. The resistance of the chitinase gene to *B. cinerea* infection was studied. In addition, *MnChi18* was heterologous expressed in *Arabidopsis* to study its function. Diverse approaches were used to study the resistance of transgenic *Arabidopsis* to *B. cinerea*, confirming that the *MnChi18* gene is involved in the defense mechanism of transgenic plants. These findings may provide effective genetic resources for improving mulberry resistance to *B. cinerea*.

## 2. Materials and Methods

### 2.1. Identification of Chitinase Genes in Mulberry

In order to identify the mulberry chitinase genes, genome sequence and annotation data were obtained from the *Morus notabilis* genome project [30]. The Hidden Markov Model (HMM) seed profiles of Glyco_hydro_18 (PF00704) and Glyco_hydro_19 (PF00182) from the Pfam database were downloaded [31]. HMMER3 (v.3.0) software was used to identify the mulberry chitinase gene [32]. Then, the presence of conserved domains of Glyco_hydro_18 or Glyco_hydro_19 was manually performed on all predicted chitinase genes.

### 2.2. Phylogenetic Tree of Chitinase Genes

To study the evolutionary relationship, ClustalW was used to align the full-length amino acid sequence of the chitinase protein under default settings, and we used MEGA6 to construct a neighbor-joining (NJ) phylogenetic tree [33]. Bootstrap analysis was performed with 1000 replicates.

### 2.3. Quantitative Real-Time PCR

Total RNA was isolated from the samples with TRIzol reagent (Invitrogen, Carlsbad, CA, USA). The QuantiNova™ SYBR Green PCR kit (Qiagen, Hilden, Germany) and StepOnePlus™ Real-time PCR system (Applied Biosystems, Waltham, MA, USA) were used for qRT-PCR detection. *AtActin* and *MnActin* genes were used as internal control genes in *Arabidopsis* and mulberry, respectively. The qRT-PCR test performed three biological replicates. Details of the qRT-PCR primers are shown in Table S1.

### 2.4. Plasmid Construction and Plant Transformation

Under the control of the CaMV35S promoter, full-length coding sequences of *MnChi18* (GenBank accession number: EXB55192.1) were cloned into the *Kpn*I (5′-GGGTACCATGGCCTCTCCCAATCCAA-3′) and *Sal*I (5′-GCGTCGACTTAGCAAGTGAGATTGGATCCA-3′) restriction sites of the pLGNL vector. Afterwards, the *CaMV35S::MnChi18* recombinant plasmid was obtained. The recombinant pLGNL expression vector was transformed into *Agrobacterium tumefaciens* strain GV3101. *MnChi18* was finally transferred into *Arabidopsis* (Columbia-0) by the floral dip method [34]. The homozygous lines of the T_3_ generation were studied.

### 2.5. Resistance Analysis of Transgenic Arabidopsis to B. cinerea

The resistance test was used to detect the ability of transgenic *Arabidopsis* plants to resist *B. cinerea*. Transgenic seeds were germinated on 1/2 Murashige and Skoog (MS) agar medium. Seven-day-old seedlings were transferred to pots of nutrient soil and grown at 24 °C/22 °C under a 16-h light/8-h dark photoperiod. The hyphal fragments were placed on the leaves of 21-day-old plants. The inoculated *Arabidopsis* plants were observed every 12 hours and photographed 36 hours later. The transgenic *Arabidopsis* plants with pLGNL were used as a control. The content of malondialdehyde (MDA) and the activity of catalase (CAT) were determined with a Malondialdehyde Assay Kit (Solarbio, Beijing, China) and Catalase Assay Kit (Solarbio, Beijing, China) according to the manufacturer’s instructions. All treatments were repeated three times.

The contents of superoxide radical (O_2_^−^) and hydrogen peroxide (H_2_O_2_) in leaves were determined by histochemical staining. The leaves were inserted into 0.1% nitroblue tetrazole (NBT) containing a 50 mM potassium phosphate buffer (pH 7.8) for O_2_^−^ detection [35]. For the detection of H_2_O_2_, a 3,3′-diaminobenzidine (DAB) solution was used in agro-infiltrated leaves [36]. The samples were placed in a 1.0 mg/mL DAB-HCl solution, darkly covered for 12 h at room temperature, and then placed in 95% ethanol for 5 minutes until brown spots of H_2_O_2_ and blue O_2_^−^ precipitate appeared on the leaves.

### 2.6. Statistical Analyses

All data were calculated using SPSS 26.0 statistical software (SPSS Inc., Chicago, IL, USA) and Excel 2013 (Microsoft, Redmond, CA, USA). The results are expressed as the mean ± standard error. Significant differences (*P* < 0.05) were measured by Student’s *t*-test analysis.

## 3. Results

### 3.1. Genome-Wide Identification and Phylogenetic Analysis of Chitinase Genes in Mulberry

A total of 26 chitinase genes were identified from the mulberry genome sequence, among which 15 belonged to the GH18 subfamily (7 Class III and 8 Class V) and 11 belonged to the GH19 subfamily (3 Class I, 4 Class II and 4 Class IV) (Table 1). The 26 predicted chitinase proteins ranged in length from 104 (*MnChi15*) to 881 amino acids (aa) (*MnChi23*). The relative molecular mass ranged from 11.78 kDa (*MnChi15*) to 96.77 kDa (*MnChi23*). The theoretical isoelectric points (pI) ranged from 4.56 (*MnChi16*) to 8.77 (*MnChi19*).

**Figure 1:**
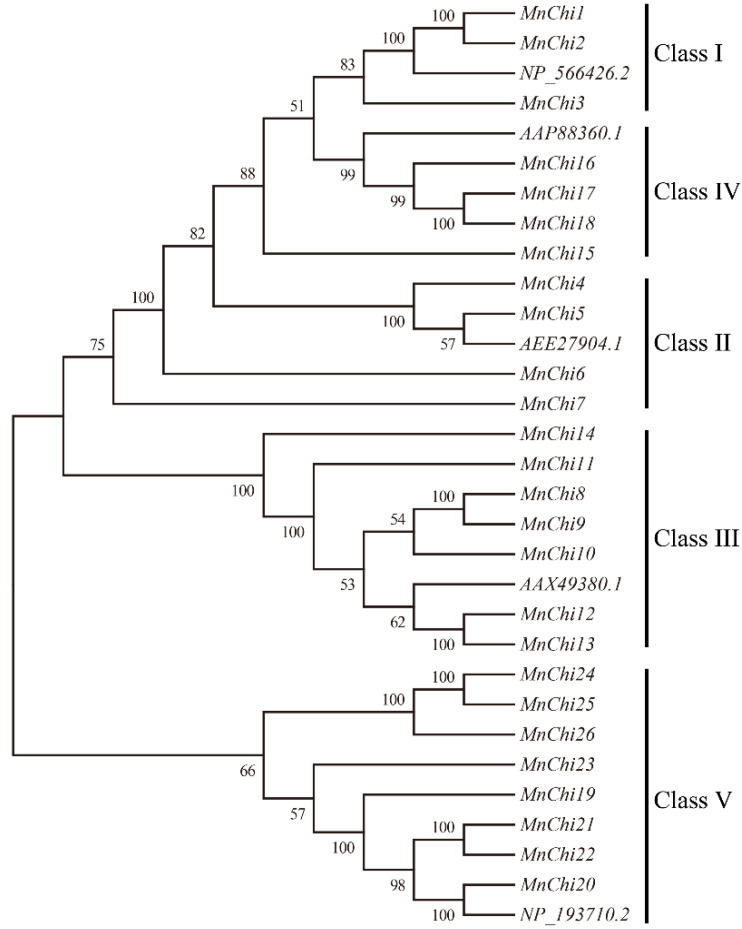
Phylogenetic tree of the chitinase genes from *M. notabilis* and *Arabidopsis*. The neighbor-joining (NJ) was used to construct a phylogenetic tree. The tree was generated by chitinase amino acid sequences using MEGA6. The numbers represent confidence percentages.

The phylogenetic analysis of the 31 chitinase sequences was carried out using the neighbor-joining method (Figure 1), and five types of chitinase proteins were identified, which was consistent with the previous *Ammopiptanthus nanus* chitinases [37]. The mulberry chitinase genes were divided into two large branches: one was composed of Classes I, II and IV, and the other was composed of Classes III and V.

### 3.2. Expression Pattern of Mulberry Chitinases under the Infection of B. cinerea

**Figure 2:**
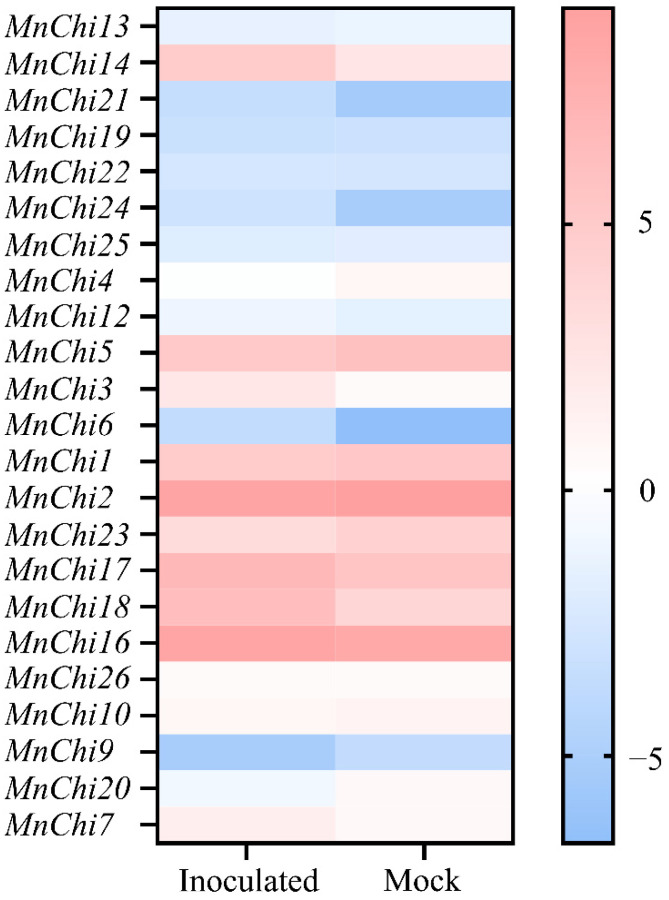
Heat map representation of the chitinase gene expression in mock-treated (Mock) and *B. cinerea*-inoculated (Inoculated) *M. notabilis* leaves. Log2 (RPKM) was used to convert the expression data to calculate the gene expression levels. The difference in gene expression was indicated by the color on the scale.

**Figure 3:**
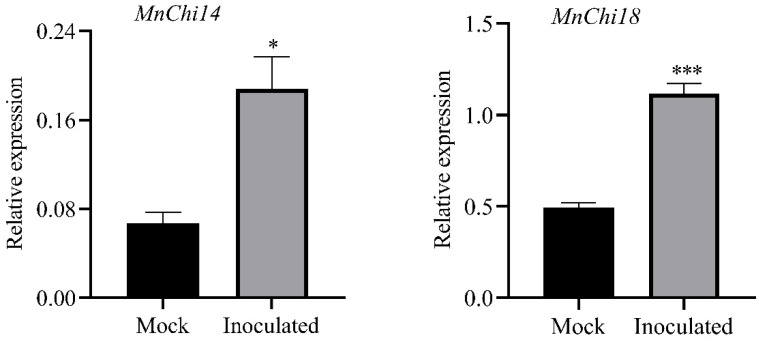
Relative expression of *MnChi14* and *MnChi18* in mock-treated (Mock) and *B. cinerea*-inoculated (Inoculated) *M. notabilis* leaves. Error bars indicate the standard deviation, *n* = 3 (* *p*-value < 0.05, *** *p*-value < 0.001; two-tailed *t*-test).

To study the resistance of mulberry chitinase genes to *B. cinerea*, we analyzed the expression pattern of chitinases based on our previous transcriptome data of mock-treated (Mock) and *B. cinerea*-inoculated (Inoculated) *M. notabilis* leaves [29]. With FPKM > 1.0, a total of 14 *MnChis* were found to be expressed (Figure 2 and Table S2). With |Fold Change (Inoculated/Mock)| > 2, the expression of four chitinases (*MnChi3/14/17/18*) in mulberry leaves was significantly upregulated after *B. cinerea* infection, and the expression of two chitinases (*MnChi20/23*) was significantly downregulated. These highly expressed chitinase genes suggested that they may be involved in mulberry resistance to *B. cinerea*. *MnChi14* and *MnChi18* were the two genes with the most increased expression after infection of *B. cinerea*, and they may play an important role in resistance to *B. cinerea* infection. Then, the two genes were verified by qRT-PCR, and the results were consistent with the transcriptome data (Figure 3). 

### 3.3. Ectopic Expression of MnChi18 in Arabidopsis Enhances Resistance to B. cinerea

**Figure 4:**
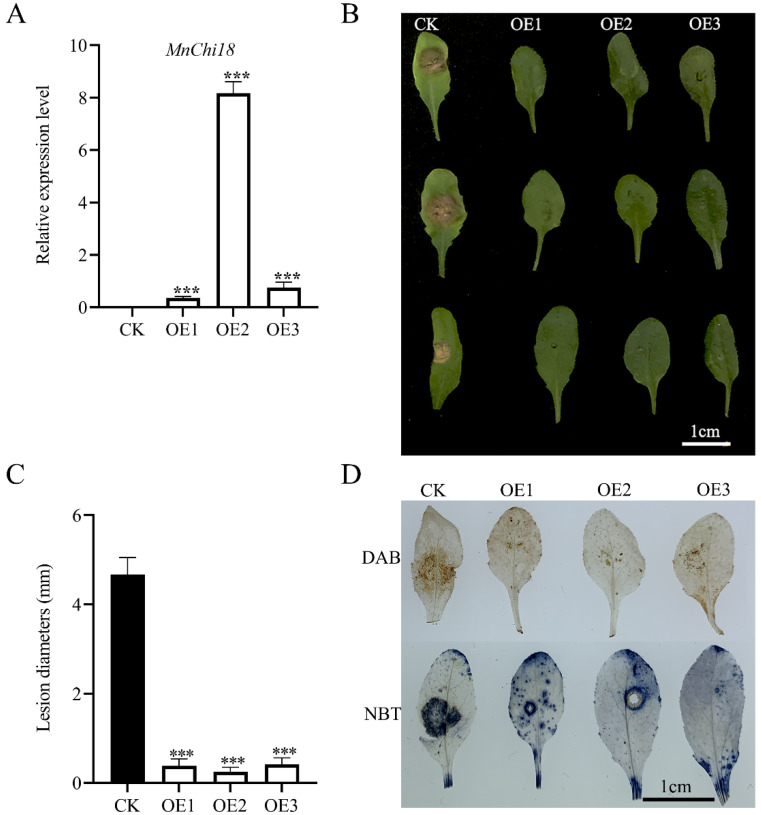
Resistance of transgenic *Arabidopsis* to *B. cinerea*. (**A**) Relative expression level of *MnChi18* in transgenic *Arabidopsis* leaves. (**B**) The leaves of *Arabidopsis* were photographed for 36 hours after being infected by *B. cinerea*. (**C**) Quantitative analysis of resistance of the empty vector transgenic (CK) and *MnChi18* transgenic (OE) lines infected by *B. cinerea*. (**D**) DAB and NBT staining revealed H_2_O_2_ and O_2_^−^ enrichment, respectively. Values are the average of three replicates. Error bars indicate SDs; *** *p*-value < 0.001.

Under the control of *Cauliflower mosaic virus* (CaMV) 35S promoter, *Arabidopsis* plants were transformed with *MnChi18* cDNA, and several T_3_ transgenic lines were obtained. The *MnChi18* gene expression was confirmed by analysis of transgenic *Arabidopsis* plants (Figure 4A). Three lines with significantly higher expression than the empty vector control were selected for follow-up study. To investigate the resistance of transgenic *Arabidopsis* with the *MnChi18* gene to *B. cinerea*, the leaves of the transgenic *Arabidopsis* with an empty vector and *MnChi18* were inoculated with an agar block containing *B. cinerea* hyphae (Figure 4B). Compared with the empty vector control leaves that showed severe disease symptoms 36 hours after inoculation, all the leaves of the *MnChi18* overexpressed lines showed only slight lesions. Quantitative analysis showed that transgenic *Arabidopsis* with *MnChi18* gene inhibited the growth of *B. cinerea* compared with transgenic *Arabidopsis* with the empty vector (Figure 4C). In addition, the production of reactive oxygen species (ROS) is the response of plants to stress. The DAB and NBT staining methods were used to detect the hydrogen peroxide (H_2_O_2_) and superoxide (O_2_^−^) in leaves, respectively. In terms of phenotype, *Arabidopsis* transferred with empty vector showed large patches of dark brown after DAB staining, an indication of H_2_O_2_ accumulation, and large patches of dark blue after NBT staining, a marker for O_2_^−^, compared with the *MnChi18* transgenic *Arabidopsis* (Figure 4D).

### 3.4. Detection of Biochemical Indices

**Figure 5:**
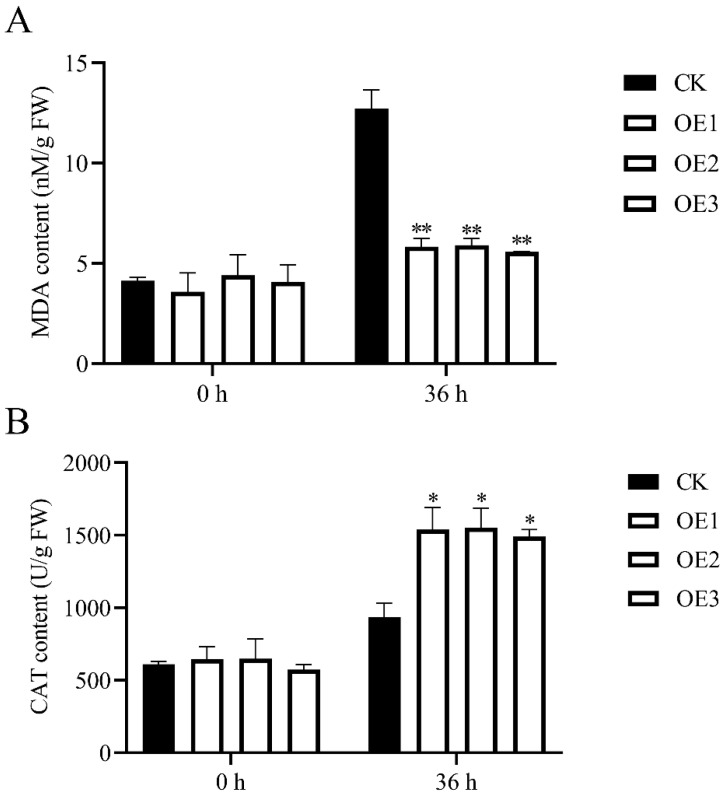
Determination of physicochemical indexes in *B. cinerea*-inoculated. (**A**) Malondialdehyde (MDA) content. (**B**) Catalase (CAT) activity. CK, empty vector transgenic plant; OE, *MnChi18* transgenic plant. Values are the average of three replicates. Error bars indicate the standard deviation; * *p*-value < 0.05 and ** *p*-value < 0.01.

In order to verify the physiological changes of transgenic *Arabidopsis* with *MnChi18* and the empty vector, the MDA content and CAT activity were determined (Figure 5). Before *B. cinerea* infection, there was no significant difference in the MDA content of transgenic *Arabidopsis* with *MnChi18* and the empty vector. After 36 h of *B. cinerea* infection, the content of MDA in both *MnChi18* and empty vector transgenic plants increased, while the content of MDA in empty vector transgenic plants was significantly higher than that in *MnChi18* transgenic plants (Figure 5A). These results suggested that plasma membrane damage was more serious in the empty vector transgenic plants than in the *MnChi18* transgenic plants. Similarly, there was no significant difference in the CAT activity of transgenic *Arabidopsis* with *MnChi18* and the empty vector before the infection of *B. cinerea*. After 36 h of *B. cinerea* infection, the CAT activity of both *MnChi18* and the empty vector transgenic plants increased, and the CAT activity of the *MnChi18* transgenic plants was significantly higher than that of the empty vector transgenic plants (Figure 5B). These results suggested that the *MnChi18* transgenic plants are more resistant to oxidative damage.

### 3.5. The Enhanced Expressions of Resistance-Related Genes in MnChi18 Transgenic Plants

**Figure 6:**
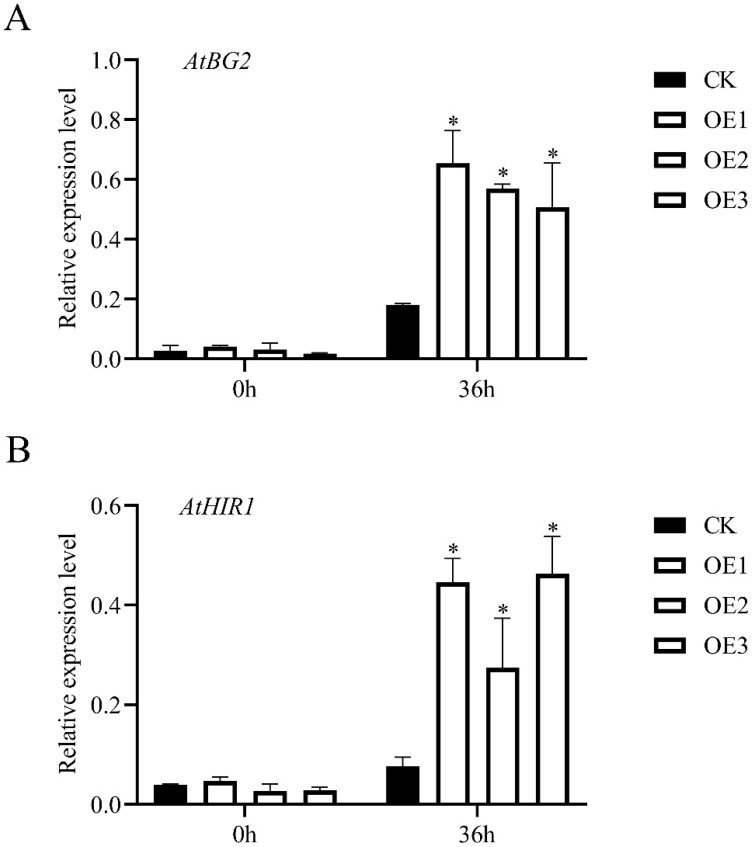
Relative expression of pathogen-related genes in the empty vector transgenic (CK) and *MnChi18* transgenic (OE) *Arabidopsis* leaves before and after of *B. cinerea* inoculation. (**A**) *AtBG2* relative expression levels; (**B**) *AtHIR1* relative expression levels. Error bars indicate the standard deviation, *n* = 3; * *p*-value < 0.05.

*PR1*, *WRKY33*, β-1,3-glucanase 2 (*BG2*) and hypersensitive induced reaction 1 (*HIR1*) are the defense-associated marker genes of a plant. The results showed that there was no significant difference between *AtPR1* and *AtWRKY33* in transgenic *Arabidopsis* with *MnChi18* and the empty vector before and after *B. cinerea* infection (Figure S1). *AtBG2* and *AtHIR1* had no significant difference in transgenic *Arabidopsis* with *MnChi18* and the empty vector before the infection of *B. cinerea*. However, the expression levels of *AtBG2* and *AtHIR1* were upregulated in both *MnChi18* and the empty vector transgenic plants after 36 h of *B. cinerea* infection, and the *MnChi18* transgenic plants were significantly higher than the empty vector transgenic plants (Figure 6). These results indicated that when the *MnChi18* gene was introduced into *Arabidopsis*, it could resist the infection of *B. cinerea* by inducing the expression of resistance-related genes.

## 4. Discussion

Chitinase genes are a large gene family, which play an important role in plant resistance. Clarifying the function of chitinase genes in plants is of great significance to plant-resistance breeding. So far, systematic genome-wide investigations of chitinase genes have been reported in many species. However, there is no systematic research report on mulberry chitinase genes. We identified a chitinase gene family in *M. notabilis* (Table 1 and Figure 1), for which a total of 26 mulberry chitinase genes were identified. Compared with the number of chitinase genes in other plants, mulberry is relatively small, but more than *Arabidopsis* [37]. This indicates that during the evolution process, the chitinase genes of mulberry have not been significantly amplified.

PR genes, including chitinase, are silenced or constitutively expressed at low levels in plants in the absence of pathogens, but are significantly induced in the presence of pathogens [38,39,40]. Consistent with previous reports, the expression of *MnChis* were both constitutive and inducible (Figure 2 and Table S2). The results showed that at least six *MnChi* genes can be induced after inoculation with *B. cinerea*. Class I (*MnChi3*), III (*MnChi14*) and IV (*MnChi17/18*) were significantly upregulated, Class II had no significantly induced expression, and Class V (*MnChi20/23*) was significantly down regulated. These findings indicate that the mulberry chitinase genes may have a different mechanism of action. Interestingly, the expression pattern of Class V was opposite to that of other plants [8,41], suggesting that the Class V chitinases in mulberry may have evolved different functions, which needs to be further studied.

To verify the function of the *MnChi18* gene, we overexpressed this gene in *Arabidopsis*. Overexpressed *MnChi18* plants were inoculated with *B. cinerea*, and ROS activity was detected by DAB and NBT staining (Figure 4). Compared with the empty vector plants, the plants overexpressing *MnChi18* had less leaf damage and ROS accumulation, thus enhancing the resistance of *Arabidopsis* leaves to *B. cinerea* infection. Our results were consistent with previous studies that *CaChiIV1* gene interference in peppers significantly reduces its resistance [42]. *MnChi18* may indirectly participate in the defense mechanism of transgenic plants by changing the transcription of other PR genes (Figure 6). Plant β-1,3-Glucanases (BG) are members of the PR2 family and one of the 17 PR protein families. It plays a key role in the response to biotic and abiotic stress. Overexpression of maize BG gene *ZmGns* in *Arabidopsis* can significantly increase the resistance to *B. cinerea* [43]. *Arabidopsis* hypersensitive-induced reaction (*AtHIR*) protein plays an important in plant innate immunity. Overexpression of *AtHIR1* inhibited the growth of *Pto* DC3000 [44]. Overexpression of *MnChi18* changes the expression of defense-related genes (*BG2* and *HIR1*), which indicates that there is an interaction between them. Overexpression of the chitinase gene can usually increase the expression of the *PR1* gene to enhance resistance [18,45], but overexpression of *MnChi18* did not enhance the expression of *AtPR1* (Figure S1), indicating that the mulberry chitin gene may have different mechanisms in plant resistance.

MDA is an important lipid peroxidation product involved in defense signal transduction in plants under biotic and abiotic stress [46]. However, our results suggested that overexpression of *MnChi18* resulted in decreased MDA accumulation (Figure 5A). The MDA levels are usually associated with oxidative stress in plants. Therefore, overexpression of *MnChi18* gene avoids cell membrane damage. When plants are invaded by pathogenic microorganisms, it will cause the accumulation of ROS and the activation of plant defense enzymes, which help maintain cell integrity and eliminate peroxides [47]. After 36 hours of infection by *B. cinerea*, the CAT activity of *MnChi18* transgenic *Arabidopsis* was significantly higher than that of the empty vector transgenic *Arabidopsis* (Figure 5B). This indicates that the overexpression of *MnChi18* in *Arabidopsis* increases the ability to maintain cell integrity and thus resist *B. cinerea* infection.

## 5. Conclusions

This study identified three Class I, four Class II, seven Class III, four Class IV and eight Class V chitinase genes from the *M. notabilis* genome sequence. The ectopic expression of *MnChi18* in *Arabidopsis* increased its resistance to *B. cinerea*, and the disease symptoms were lighter. Overexpression of *MnChi18* protected plant cells from damage and enhanced the expression of plant resistance genes. This study will provide basic insights into the role of the *MnChi18* gene in the resistance pathway.

## Figures and Tables

**Table 1:** Characterization of the chitinases in *M. notabilis*.

Gene Name	Gene ID	Class	Domains	GenBank Acc.	CDS (bp)	Size (aa)	MW (kDa)	Predicted pI
*MnChi1*	*L484_014360*	I	GH19	EXB95387.1	978	325	34.73	7.80
*MnChi2*	*L484_014362*	I	GH19	EXB95389.1	978	325	34.89	7.38
*MnChi3*	*L484_013887*	I	GH19	EXB44469.1	762	253	27.84	6.42
*MnChi4*	*L484_007737*	II	GH19	EXB55741.1	960	319	35.25	6.78
*MnChi5*	*L484_012010*	II	GH19	EXB97442.1	957	318	35.10	6.97
*MnChi6*	*L484_014359*	II	GH19	EXB95386.1	627	208	21.98	6.30
*MnChi7*	*L484_026587*	II	GH19	EXC35265.1	1032	343	37.92	6.44
*MnChi8*	*L484_022481*	III	GH18	EXB52704.1	900	299	32.10	6.50
*MnChi9*	*L484_022482*	III	GH18	EXB52705.1	897	298	32.01	5.36
*MnChi10*	*L484_020224*	III	GH18	EXB97674.1	1527	508	54.85	5.26
*MnChi11*	*L484_011484*	III	GH18	EXB72482.1	630	209	22.97	6.55
*MnChi12*	*L484_011486*	III	GH18	EXB72483.1	903	300	32.71	8.65
*MnChi13*	*L484_000037*	III	GH18	EXC45568.1	600	199	22.08	7.66
*MnChi14*	*L484_000761*	III	GH18	EXC37464.1	2448	815	91.33	7.95
*MnChi15*	*L484_022490*	IV	GH19	EXB52713.1	315	104	11.78	7.88
*MnChi16*	*L484_018124*	IV	GH19	EXB55197.1	840	279	30.33	4.56
*MnChi17*	*L484_018118*	IV	GH19	EXB55191.1	825	274	29.53	4.59
*MnChi18*	*L484_018119*	IV	GH19	EXB55192.1	825	274	29.42	4.71
*MnChi19*	*L484_003149*	V	GH18	EXC13800.1	1269	422	46.72	8.77
*MnChi20*	*L484_022978*	V	GH18	EXB94872.1	1101	366	40.55	5.11
*MnChi21*	*L484_001056*	V	GH18	EXB47196.1	2121	706	79.36	8.10
*MnChi22*	*L484_003690*	V	GH18	EXC19668.1	2304	767	87.03	8.40
*MnChi23*	*L484_017594*	V	GH18	EXB62207.1	2646	881	96.77	6.56
*MnChi24*	*L484_007185*	V	GH18	EXB53242.1	936	311	34.89	6.35
*MnChi25*	*L484_007186*	V	GH18	EXB53243.1	909	302	33.99	7.79
*MnChi26*	*L484_020088*	V	GH18	EXB80831.1	822	273	30.56	5.96

## Data Availability

The datasets supporting the conclusions of this article are included within the article.

## Supplementary Materials

The following are available online at https://www.mdpi.com/article/10.3390/genes13010098/s1: Figure S1: Relative expression of pathogen-related genes in empty vector transgenic (CK) and MnChi18 transgenic (OE) Arabidopsis leaves before and after of B. cinerea inoculation; Table S1: Primers for real-time PCR; Table S2: Differential expression analysis of chitinase gene in Mock and Inoculated.
