# Supplementary material for: Characterization of the Chitinase Gene Family in Mulberry (Morus notabilis) and MnChi18 Involved in Resistance to Botrytis cinerea
Source: Genes (Basel). 2021 Dec 31;13(1):98. doi: 10.3390/genes13010098 (PMC8774697; doi:10.3390/genes13010098)
Supplement: Supplementary file 1 [file genes-13-00098-s001.zip › genes-1519354-supplementary.pdf]

Table S1. Primers for real-time PCR.

| Gene symbol | Forward primer | Reverse primer |
|---|---|---|
| *MnChi14* | TACAAGATGCGAGGGGA | GCAAGCCGAAATGACTG |
| *MnChi18* | AACGCTGCCAACGCCTACCCT | TCTTTGCCGTCACATTCCACA |
| *AtBG2* | TAAGAGCTTTCTCGAACCAG | AGTACCCTGGATCGTTATCA |
| *AtHIR1* | CATGGTGTCTTGGTAGTCAA | AATCTGACCCCTTGTGTTAC |

Table S2. Differential expression analysis of chitinase gene in Mock and Inoculated.

| Gene ID | Inoculated FPKM | Mock FPKM | log2(Inoculated/Mock) |
|---|---|---|---|
| *MnChi13* | 0.357 | 0.430 | -0.269760269 |
| *MnChi14* | 30.127 | 5.323 | 2.500639244 |
| *MnChi21* | 0.087 | 0.023 | 1.893084796 |
| *MnChi19* | 0.110 | 0.113 | -0.043068722 |
| *MnChi22* | 0.170 | 0.157 | 0.11783649 |
| *MnChi24* | 0.127 | 0.027 | 2.247927513 |
| *MnChi25* | 0.263 | 0.290 | -0.139162748 |
| *MnChi4* | 0.917 | 1.707 | -0.896712192 |
| *MnChi12* | 0.480 | 0.320 | 0.584962501 |
| *MnChi5* | 34.940 | 60.497 | -0.791976045 |
| *MnChi3* | 4.800 | 1.387 | 1.791413378 |
| *MnChi6* | 0.077 | 0.010 | 2.938599455 |
| *MnChi1* | 30.283 | 39.787 | -0.393761021 |
| *MnChi2* | 400.970 | 533.907 | -0.413093263 |
| *MnChi23* | 9.910 | 20.540 | -1.051479219 |
| *MnChi17* | 108.427 | 44.490 | 1.285166616 |
| *MnChi18* | 78.163 | 14.203 | 2.460262443 |
| *MnChi16* | 378.167 | 292.463 | 0.37076644 |
| *MnChi26* | 1.360 | 1.347 | 0.014213859 |
| *MnChi10* | 1.713 | 2.147 | -0.325292329 |
| *MnChi9* | 0.027 | 0.080 | -1.584962501 |
| *MnChi20* | 0.530 | 1.600 | -1.59400764 |
| *MnChi7* | 3.103 | 1.657 | 0.905535316 |

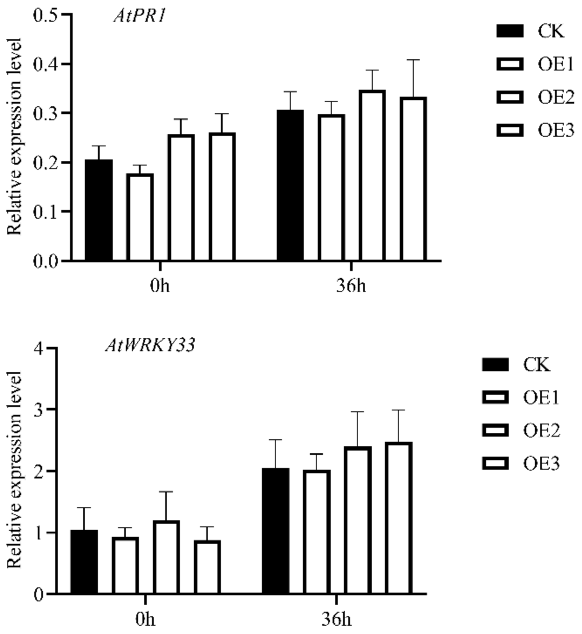


**Figure S1.** Relative expression of pathogen-related genes in empty vector transgenic (CK) and *MnChi18* transgenic (OE) *Arabidopsis* leaves before and after of *B. cinerea* inoculation. (A) *AtPR1* relative expression levels; (**b**) *AtWRKY33* relative expression levels. Error bars indicate standard deviation, n = 3, **P*-value < 0.05.
